# Minimal Associations between Short-Term Dietary Intake and Salivary Microbiome Composition

**DOI:** 10.3390/microorganisms9081739

**Published:** 2021-08-15

**Authors:** Judith Kim, Minyi Lee, Brittany Baldwin-Hunter, Quinn S. Solfisburg, Charles J. Lightdale, Tal Korem, Chin Hur, Julian A. Abrams

**Affiliations:** 1 Department of Medicine, Columbia University Irving Medical Center, New York, NY 10032, USA; jk3848@cumc.columbia.edu (J.K.); brb2025@nyp.org (B.B.-H.); cjl18@cumc.columbia.edu (C.J.L.); ch447@cumc.columbia.edu (C.H.); 2 School of Medicine, Boston University, Boston, MA 02118, USA; minyilee@bu.edu; 3 Vagelos College of Physicians and Surgeons, Columbia University Irving Medical Center, New York, NY 10032, USA; quinnsolfisburg@gmail.com; 4 Program for Mathematical Genomics, Department of Systems Biology, Columbia University, New York, NY 10032, USA; tk2829@cumc.columbia.edu

**Keywords:** salivary microbiome, esophageal cancer, dietary intake, esophageal microbiome

## Abstract

Background: Increasing evidence points to the esophageal microbiome as an important co-factor in esophageal neoplasia. Esophageal microbiome composition is strongly influenced by the oral microbiome. Salivary microbiome assessment has emerged as a potential non-invasive tool to identify patients at risk for esophageal cancer, but key host and environmental factors that may affect the salivary microbiome have not been well-defined. This study aimed to evaluate the impact of short-term dietary intake on salivary microbiome composition. Methods: Saliva samples were collected from 69 subjects prior to upper endoscopy who completed the Automated Self-Administered 24-Hour (ASA24) Dietary Assessment. Salivary microbiome composition was determined using 16S rRNA amplicon sequencing. Results: There was no significant correlation between alpha diversity and primary measures of short-term dietary intake (total daily calories, fat, fiber, fruit/vegetables, red meat intake, and fasting time). There was no evidence of clustering on beta diversity analyses. Very few taxonomic alterations were found for short-term dietary intake; an increased relative abundance of *Neisseria oralis* and *Lautropia sp.* was associated with high fruit and vegetable intake, and an increased relative abundance of a taxon in the family *Gemellaceae* was associated with increased red meat intake. Conclusions: Short-term dietary intake was associated with only minimal salivary microbiome alterations and does not appear to have a major impact on the potential use of the salivary microbiome as a biomarker for esophageal neoplasia.

## 1. Introduction

The esophageal microbiome is broadly similar to the oral microbiome, with an abundance of anaerobes and a high Firmicutes to Bacteroidetes ratio [1,2]. This link between the mouth and esophagus was demonstrated by a recent, randomized controlled trial of an antimicrobial mouth rinse, in which oral microbiome alterations were found to directly impact esophageal microbiome composition and cause marked changes in esophageal tissue gene expression [3]. In light of the oral–esophageal microbiome connection, esophageal microbiome changes associated with neoplasia may be reflected in the mouth, an easily accessible sampling site. The salivary microbiome is highly distinct in patients with Barrett’s esophagus (BE), the esophageal adenocarcinoma (EAC) precursor, and a microbiome signature distinguishes patients with and without BE with high accuracy [4]. Alterations to the oral microbiome can be detected years prior to the development of EAC [5]. The oral microbiome has also been directly and indirectly linked to esophageal squamous cell cancer (ESCC) risk. Decreased diversity has been observed in saliva and upper GI tissue from esophageal squamous dysplasia and cancer patients [6,7]. Poor oral health, poor oral hygiene, and tooth pitting, all associated with a perturbed oral microbiome, have also been associated with increased risks of ESCC [8,9]. Thus, the oral microbiome represents a logical and attractive target as a non-invasive test to identify patients at risk for esophageal cancer.

Saliva collection for oral microbiome assessment is ideally suited for translation to the clinical setting. Saliva collection is very easy, can be performed in any setting, and can be repeated longitudinally. The salivary microbiome is highly stable over time [10,11,12], especially compared to other body sites [13], and is resistant to perturbations [14]. Further, the method of saliva collection does not greatly impact microbiome composition analysis [15]. However, for the salivary microbiome to be clinically useful as a biomarker, it is critically important to understand host and environmental factors that could impact its test characteristics.

Dietary habits and interventions impact the lower gut microbiome, and diet-associated microbial alterations have been correlated with diseases such as colorectal cancer, diabetes, and obesity [16,17,18,19,20]. Acute, short-term dietary changes can profoundly alter the microbial composition of the lower gut within a few days [21]. In comparison, the association between diet, especially short-term dietary intake, and the salivary microbiome has not been extensively studied. A small number of studies have evaluated associations between longer term dietary patterns and oral microbiome composition. One recent study analyzed saliva samples from 292 Danish participants with mild dental disease and found no association between long-term dietary habits and salivary microbiome composition [22]. Another study by Kato et al. utilized a comprehensive long-term dietary survey, including micronutrients, to explore associations between usual dietary habits and the oral microbiome [23]. The authors observed that regular intake of daily saturated fatty acids and vitamin C correlated with modest changes in alpha diversity indices. 

While these studies evaluated the associations between long-term dietary habits and the salivary microbiome, dietary intake in the time period immediately preceding specimen collection was not assessed. This is an important gap in the knowledge, as effects of dietary intake shortly prior to saliva collection and analysis could impact the utility of the salivary microbiome as a clinical biomarker for esophageal neoplasia. In this study, we aimed to evaluate the associations between short-term dietary intake and salivary microbiome composition.

## 2. Materials and Methods

### 2.1. Study Population

A prospective study was conducted of patients ≥18 years old who were scheduled to undergo an upper endoscopy for clinical indications. Subjects were enrolled over a 12-month period at a single academic medical center (Columbia University Irving Medical Center, New York, NY). All subjects provided written informed consent. This study was approved by the Columbia University Irving Medical Center Institutional Review Board. Subjects were excluded for concurrently scheduled colonoscopy, antibiotic, steroid, or other immunosuppressant use within the previous 3 months, and for history of gastric or esophageal surgery.

Information was collected from the participants themselves as well as from the electronic medical record, including age, sex, race, ethnicity, body mass index, smoking status, oral hygiene habits, recent dietary intake, history of cancer, and recent medication use, including antibiotics, proton-pump inhibitors, and H2 receptor antagonists. All patients were fasting since midnight on the night prior to sample collection. Patients were asked to spit into a container for saliva collection.

### 2.2. Dietary Intake

Dietary intake was assessed using the Automated Self-Administered 24-Hour (ASA24) Dietary Assessment tool (https://epi.grants.cancer.gov/asa24/; date first accessed on 31 December 2017). This tool helps patients record their dietary intake from the previous full day, capturing every food and beverage item they consumed, how it was prepared, and the quantity that was consumed. The tool uses pictures to assist in determining portion size and includes multiple prompts for frequently forgotten items, such as cooking oil or glasses of water. These detailed inputs are matched to the Food and Nutrient Database for dietary studies and then summarized in an aggregate as 65 nutrients and 37 food groups. These nutrients include macro-nutrients such as total fat and total calories, as well as vitamins, minerals, fats and fatty acids, and other substances such as caffeine. The ASA24 tool was developed by the National Cancer Institute and Westat and was modeled after the dietary interview component of the National Health and Nutrition Examination Survey [24]. All reported foods are automatically coded.

### 2.3. Microbiome Analyses

Genetic material was extracted in four batches, using Qiagen MagAttract PowerMicrobiome DNA/RNA with sequencing for hypervariable ribosomal RNA regions V3-V4 on the Illumina MiSeq 2 × 300 and using the primers 5’TCGTCGGCAGCGTCAGATGTGTATAAGAGACAGCCTACGGGNGGCWGCAG and 5’GTCTCGTGGGCTCGGAGATGTGTATAAGAGACAGGACTACHVGGGTATCTAATCC. Patient sequences were processed using the Bioconductor package *dada2* (Version 1.16), using the standard pipeline to construct an Amplicon Sequence Variant (ASV) table. Taxonomy was assigned with a ≥ 97% cutoff against the Greengenes version 13.8 reference dataset. Downstream analysis was performed using the R package, *phyloseq* (Version 1.32.0). Before any diversity analysis was performed, the data were subsampled to a sample size of 10,000. Five samples were removed in this process because they contained too few reads. 

Alpha diversity was evaluated using Shannon and Chao1 indices. Beta diversity analysis was performed using Bray–Curtis for distance and was visualized through principal coordinates analysis (PCoA). Groups were compared using permutational ANOVA (PERMANOVA) analysis. To test for differences in abundance at the ASV level, the R package *DESeq2* (Version 1.28.1) was used. Differential abundances were corrected for multiple comparisons using the Benjamini–Hochberg procedure, and corrected statistical significance was defined as *p* < 0.05.

### 2.4. Statistical Analysis

Continuous variables were summarized using medians and categorical variables were summarized using proportions. Categorical variables were compared between groups using Fisher’s exact tests, and continuous variables were compared using Wilcoxon rank-sum tests. Spearman-correlation coefficients were calculated to assess for correlations between alpha diversity and dietary intake components. Samples for each nutrient component were grouped into quartiles, and indices in the first and fourth quartile were compared using Wilcoxon rank-sum tests. The only exception was red meat, which was stratified into two groups above and below the median. It could not be divided into quartiles, as more than one-quarter of the subjects had not consumed any red meat. Multivariable linear regression models were built to assess the effect of total daily calories, fat, fiber, fruit/vegetables, and red meat on the associations between significant clinical variables and alpha diversity. *p*-values less than 0.05 were considered statistically significant, with adjustment for multiple comparisons made using the Bonferroni method. Analyses were performed using R statistical software (Version 4.0.2). 

## 3. Results

### 3.1. Study Population Characteristics

A total of 69 participants met the inclusion criteria, completed the dietary intake survey, and had complete sequencing data for analysis. Baseline characteristics of the study population are shown in Table 1. The median age was 61 years and 65% of subjects were male. The median BMI was 26.6. Almost all brushed their teeth daily or more often (95%), whereas only 28% used mouthwash daily. The indications for upper endoscopy are shown in Supplementary Table S1. 

A summary of dietary intake on the day prior to sample collection is shown in Table 2 and Supplementary Table S2. Median daily total energy was 1698 kcal (IQR 1268–2302), median daily fat intake was 72.7 g (IQR 40.2–96.8), median daily fiber intake was 14.5 g (IQR 9.5–20.9), median intake of daily fruits and vegetables was 1.8 cups (IQR 0.8–3.0), and median daily red meat consumption was 0.98 ounces (IQR 0–2.8). The median fasting time was 12 h prior to saliva collection (IQR 11–13). 

### 3.2. Salivary Microbiome and Dietary Intake

**Figure 1:**
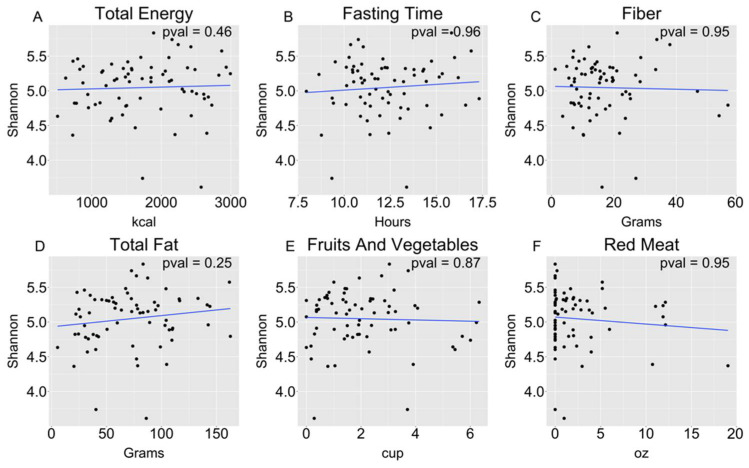
There were no significant correlations between short-term dietary intake measures and alpha diversity: (**A**) total energy; (**B**) fasting time; (**C**) fiber; (**D**) total fat; (**E**) fruits and vegetables; (**F**) red meat. Unadjusted *p*-values are shown.

**Figure 2:**
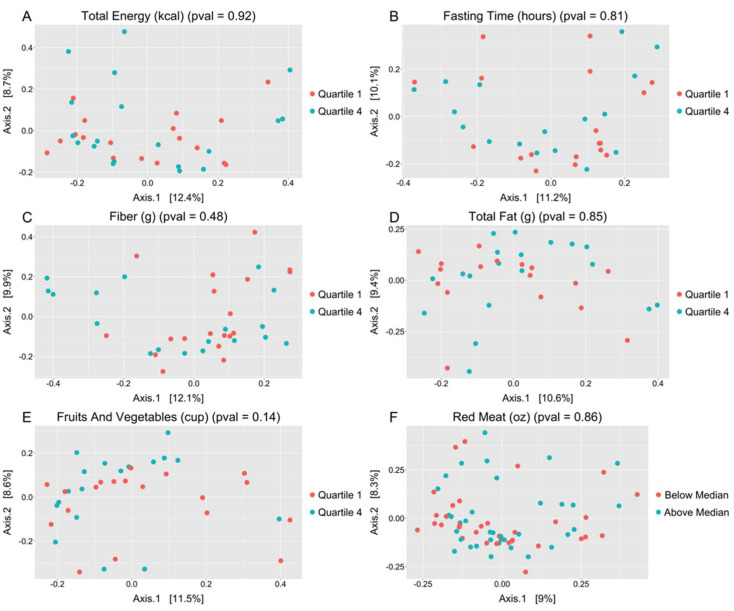
There was no evidence of significant clustering on beta diversity analyses by Bray–Curtis dissimilarity (PERMANOVA) of lowest and highest quartiles of short-term dietary intake measures: (**A**) total energy; (**B**) fasting time; (**C**) fiber; (**D**) total fat; (**E**) fruits and vegetables; (**F**) red meat (red meat categorized as above or below median). Unadjusted *p*-values are shown.

There were no significant correlations between alpha diversity and any of the primary measures of dietary intake, or between alpha diversity and fasting time (Figure 1). There were also no significant correlations between alpha diversity and the other nutrients assessed (Supplementary Figure S1). Clustering was not detected within beta diversity analyses based on measures of short-term dietary intake (Figure 2). There were surprisingly few differentially abundant taxa in the various comparisons of dietary intake (Supplementary Table S3). In fact, in analyses of the major dietary intake components, the only differences found were increases in *Neisseria oralis* and *Lautropia sp.*, associated with the highest quartile of fruit and vegetable intake, and an increased relative abundance of a taxon in the family *Gemellaceae*, associated with above median red meat intake. Interestingly, increased *N. oralis* was also associated with an increased intake of vitamin B12, vitamin C, and sugars.

### 3.3. Salivary Microbiome and Clinical Features

There was a non-significant positive correlation between salivary microbiome alpha diversity and BMI (ρ  = 0.28; *p* = 0.02, adjusted *p* = 0.18). Subjects taking histamine-2-receptor agonist (H2RA) had non-significant higher alpha diversity than those who were not (rank sum *p* = 0.03, adjusted *p* = 0.27) (Supplementary Figure S2). No associations or correlations were found between other clinical characteristics and alpha diversity (adjusted *p* for all > 0.36). Given these findings, multivariable linear regression analyses were performed to reassess the associations between alpha diversity and BMI, H2RA use, and components of dietary intake. The associations between BMI or H2RA use and alpha diversity were qualitatively similar when adjusting for all the major dietary variables (total daily calories, fat, fiber, fruit/vegetables, and red meat). On beta diversity analyses, there was non-significant clustering according to participant tooth loss (PERMANOVA *p* = 0.02; adjusted *p*-value 0.18). There was otherwise no evidence of clustering based on clinical features. In differential abundance analyses, older age was associated with a significantly reduced relative abundance of *N. oralis* (Supplementary Table S3). Patients who used mouthwash on the day of saliva collection had significantly higher relative abundance of *Actinomyces sp.* compared to those who did not (adjusted *p* = 0.007). Otherwise, there were no differences in the relative abundance of taxa based on the clinical characteristics evaluated in this study.

## 4. Discussion

In this study of patients undergoing upper endoscopy, there were only minimal associations between short-term dietary intake and salivary microbiome composition. There were no associations with alpha diversity analyses and only a few taxa were differentially abundant based on intake of various dietary components. Fasting time also did not have an appreciable impact on salivary microbiome composition. Interestingly, there was evidence that age and H2-receptor antagonist use may influence the salivary microbiome. Overall, these findings suggest that short-term dietary intake does not represent a major confounder of salivary microbiome analysis when used as a clinical biomarker for esophageal neoplasia.

The current study adds to the body of the literature that supports the notion that the salivary microbiome is relatively stable. In a 2018 study, Vogtmann et al. collected oral samples from 40 subjects, approximately every 2 months for 6 months, and calculated intraclass correlation coefficients for alpha diversity metrics, beta diversity metrics, and the top four most abundant phyla in the samples [10]. The study found a high intraclass correlation between alpha and beta diversity metrics, though relative abundance may be less stable. They also reported a high probability of an individual remaining within the same community type over a period of 6 months. Belstrom et al. analyzed saliva samples from 5 healthy subjects, taken at 4-hour intervals over a 48-hour period, to determine whether the composition of the salivary microbiota varied during the day. There was little or no evidence of short-term alterations to the salivary microbiome of the subjects throughout the day [11]. Though the process of food digestion begins in the mouth, this is a transitory process compared to intestinal digestion, which may explain diet’s larger impact on the lower gut microbiome. 

In a prior cross-sectional study of 49 patients with and without Barrett’s esophagus, taxa assigned to the species *Lautropia* and *Streptococcus* made up part of a salivary microbiome signature that distinguished patients with and without BE with high accuracy (AUC 0.94) [4]. This signature remained predictive of BE even after adjusting for known EAC risk factors, including BMI. In a cohort study, Peters et al. found that increased relative abundance of *Tannerella forsythia* in an oral rinse was significantly associated with future risk of EAC [5]. In the current study, short-term dietary intake was not associated with alterations in the relative abundance of these BE- and EAC-associated bacteria, although the study was not designed to address this specifically. 

Subjects with the highest quartile of fruits and vegetables intake had higher relative abundance of *Neisseria oralis* and *Lautropia sp*. *N. oralis* relative abundance also differed based on intake of vitamin B12, vitamin C, and sugars, suggesting that the metabolism of this bacteria may be particularly sensitive to dietary components. A prior study noted an increased relative abundance of *Neisseria* in saliva from vegans compared to omnivores [25]. Whether fruit and vegetable intake is a surrogate marker for better health and health habits or whether it directly impacts the relative abundance of *N. oralis* and *Lautropia sp.* in the mouth is unclear. BMI and use of H2RA were associated with differences in alpha diversity. Prior studies on the association between BMI and the salivary microbiome in adults have varied in design and have had inconsistent findings [22,26,27]. The reasons for the observed associations in the current study are unclear and would need to be replicated.

Studies of long-term dietary intake patterns have also largely failed to consistently demonstrate alterations to the oral microbiome [22,23,28,29,30]. When comparing healthy individuals in Italy who have omnivore, vegetarian, or vegan diets, which have major differences in macro- and micro-nutrient content, De Filippis et al. found that dietary habits had no significant association with the diversity and composition of salivary microbiota [28]. However, another recent study by Hansen et al. reported differentially abundant microbiota between omnivores and vegans in salivary microbiome composition, including an increased relative abundance of *Neisseria subflava* in vegans [25]. Other studies have also evaluated specific macro- and micro-nutrients and found no or minimal association with the salivary microbiome [22,23,30]. Belstrom et al. analyzed the saliva of 292 subjects, comparing those in the upper and lower quintiles of daily fat, protein, carbohydrate, and added sugar intake based on a food frequency questionnaire. They found that salivary microbiome composition was independent of dietary intake [22]. In 182 American subjects, Kato et al. also observed minimal associations between the oral microbiome and regular dietary intake, including multiple minerals and vitamins [23]. A recent large study of 1049 Canadians assessed habitual dietary intake based on a self-reported questionnaire, and also did not validate any significant associations between dietary intake and oral microbiome composition [30].

Tooth loss status was the only characteristic in the current study with evidence of clustering on beta diversity analyses. Oral dysbiosis has been associated with multiple oral and dental conditions, including oral cancer and periodontitis. One study of 2343 Japanese adults reported that salivary phylogenetic diversity was significantly correlated with number of teeth, presence of dental caries, periodontal disease, gingival bleeding, and dental plaques [26]. Bornigen et al. evaluated the oral microbiome of 121 oral cancer cases and 242 matched controls [31]. Interestingly, tooth loss status was associated with a large effect on oral microbiome composition, even greater than that of oral cancer itself. Future studies assessing the oral microbiome as a clinical biomarker will need to carefully evaluate the impact of tooth loss and periodontal disease.

The strengths of the current study include the use of a validated, comprehensive food frequency questionnaire that captured dietary intake on the day prior to sample collection, allowing for the assessment of associations between proximal dietary exposure and salivary microbiome composition. We also obtained information on oral and dental diseases and dental hygiene habits, which are known to have strong associations with the oral microbiome. The median daily dietary estimates for macronutrient intake, such as fat and fiber, in the study population were comparable to national dietary data [32]. The study population was also diverse with regard to age and sex. There were also certain limitations: Estimated dietary intakes from questionnaires may be subject to bias or recall error, although this may be less of an issue for short-term dietary recall. Some of the null associations in the study may be due to the relatively small sample size. The study population was drawn from subjects who were undergoing upper endoscopy for clinical indications, which may limit its generalizability.

In conclusion, short-term dietary intake was associated with only minimal salivary microbiome alterations. These findings are similar to prior studies demonstrating that long-term dietary patterns have minimal impact on the salivary microbiome. Together, these data support the notion that dietary intake may not be a major confounder in salivary microbiome analyses. Diet does not appear to represent a major impediment to the use of the salivary microbiome as a biomarker for esophageal neoplasia, although larger prospective studies are necessary to identify other potential host and environmental factors that may impact the clinical utility of the salivary microbiome to identify patients at risk for esophageal cancer.

## Figures and Tables

**Table 1:** Study population characteristics.

	(*n* = 69)
Age, median (IQR)	61 (42–71)
BMI, median (IQR)	26.6 (22.3–29.2)
Male sex	45 (65%)
PPI use	12 (17%)
Smoking	
Current	2 (3%)
Former	22 (32%)
Never	45 (65%)
*Oral health and hygiene*	
Tooth loss status	
Has all or most of natural teeth	44 (64%)
Has partial plates or implants	20 (29%)
Has full upper and lower dentures or implants	2 (3%)
Has full upper dentures or implants	2 (3%)
Unknown	1 (1%)
Tooth brushing	
Daily	25 (36%)
Less than daily	3 (4%)
More than daily	41 (59%)
Brush on day of collection—yes	59 (86%)
Mouthwash in the past month n (%)	
Daily	19 (28%)
Less than daily	19 (28%)
Does not use	31 (44%)
Day of collection—yes	19 (28%)

**Table 2:** Summary of dietary intake from the day prior to saliva sample collection.

Total Calories (kcal)	
Median (interquartile range)	1698 (1268–2302)
Range	508.7–2993.51
Total Fat (g)	
Median (interquartile range)	72.7 (40.2–96.9)
Range	5.5–162.6
Fiber (g)	
Median (interquartile range)	14.5 (9.5–20.9)
Range	1.2–56.6
Fruit/Veg (cups)	
Median (interquartile range)	1.8 (0.8–3.0)
Range	0.0–6.3
Red Meat (oz)	
Median (interquartile range)	0.99 (0–2.8)
Range	0–19.0
Fasting Time (hours)	
Median (interquartile range)	12 (11–13)
Range	8–17

## Data Availability

The data presented in this study are available on request from the corresponding author.

## Supplementary Materials

The following are available online at https://www.mdpi.com/article/10.3390/microorganisms9081739/s1, Figure S1: There was no evidence of significant clustering on beta diversity analyses by Bray-Curtis dissimilarity (ANOSIM) of lowest and highest quartiles of short-term dietary intake measures, Figure S2: Salivary microbiome alpha diversity associations with patient characteristics, Table S1: Indications for upper endoscopy, Table S1: Summary of additional nutrients based on dietary intake data from the day prior to saliva collection, Table S3: There were relatively few differentially abundant bacterial taxa comparing highest vs. lowest quartile of components of short-term dietary intake (above vs. below median for red meat). There were no differentially abundant taxa associated with intake of other nutrients.
