# Supplementary material for: Minimal Associations between Short-Term Dietary Intake and Salivary Microbiome Composition
Source: Microorganisms. 2021 Aug 15;9(8):1739. doi: 10.3390/microorganisms9081739 (PMC8401849; doi:10.3390/microorganisms9081739)
Supplement: Supplementary file 1 [file microorganisms-09-01739-s001.zip › microorganisms-1353213-supplementary.docx]

**Supplementary Figure 1.** There were no significant correlations between salivary microbiome alpha diversity and short-term dietary intake of additional nutrients: **A)** sodium; **B)** sugars; **C)** saturated fatty acids; **D)** cholesterol; **E)** carbohydrate; **F)** calcium; **G)** magnesium; **H)** vitamin B12; **I)** vitamin C; **J)** vitamin D. Unadjusted p-values are shown.


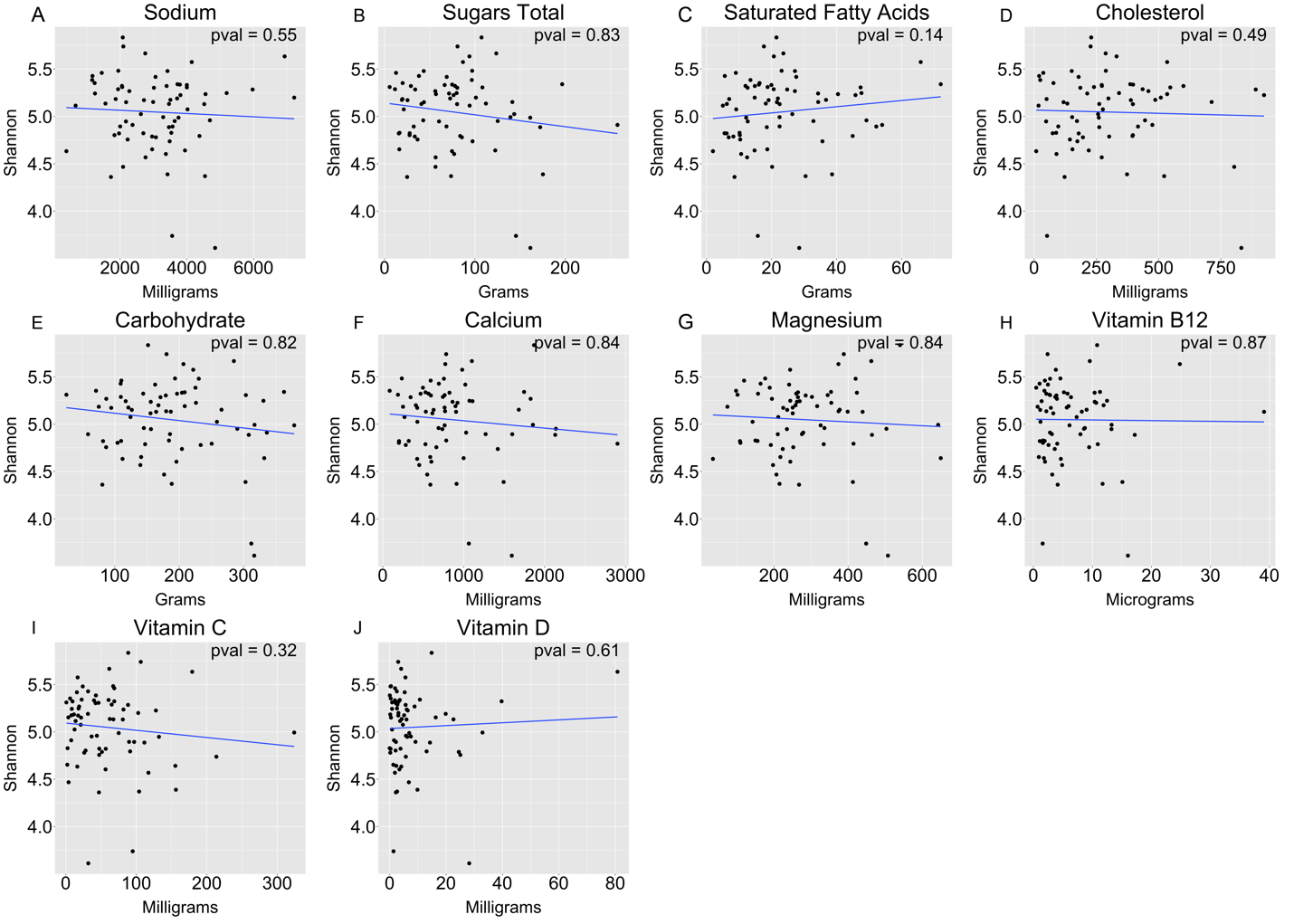


**Supplementary Figure 2.** Salivary microbiome alpha diversity associations with patient characteristics: A) age; B) BMI; C) sex; D) tooth loss; E) mouthwash use (at least daily); F) toothbrushing (at least daily); G) GERD; H) PPI use; I) H2RA use. Unadjusted p-values are shown; none of the correlations or associations was statistically significant after adjusting for multiple comparisons.


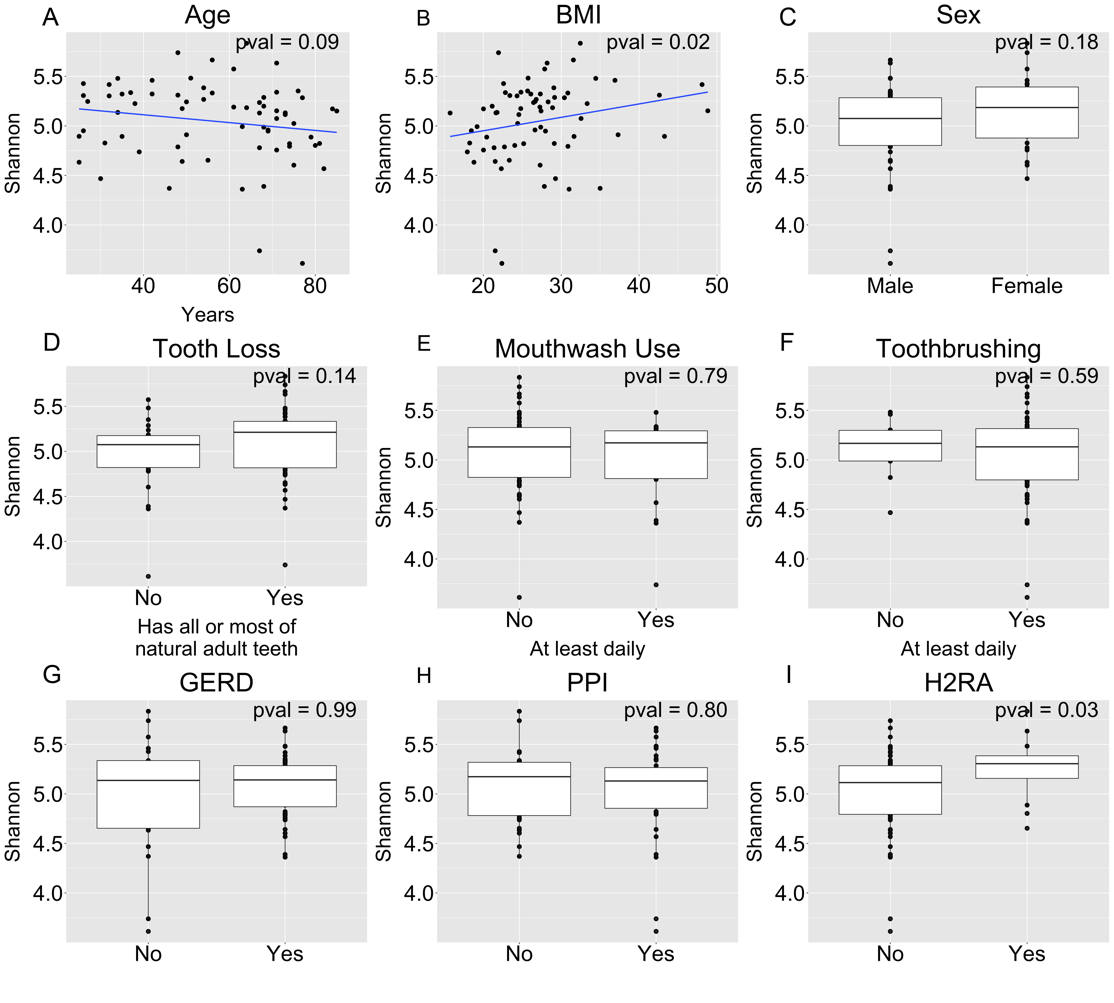


**Supplementary Table 1.** Indications for upper endoscopy.

|  | **n (%)** |
| --- | --- |
| History or suspicion of Barrett's esophagus | 29 (42.0%) |
| Abdominal pain/heartburn | 11 (15.9%) |
| Dysphagia | 7 (10.1%) |
| History of celiac disease | 15 (21.7%) |
| Other | 7 (10.1%) |

**Supplementary Table 2.** Summary of additional nutrients based on dietary intake data from the day prior to saliva collection.

| **Nutrient** | **Median (IQR)** |
| --- | --- |
| Sodium (mg) | 3061 (2081-3750) |
| Total Sugar (g) | 73.6 (33.9-97.3) |
| Saturated Fatty Acids (g) | 20.0 (12.2-28.5) |
| Cholesterol (mg) | 261.6 (151.2-397.3) |
| Carbohydrates (g) | 179.8 (124.4-230.5) |
| Calcium (mg) | 755.0 (526.4-1029.4) |
| Magnesium (mg) | 259.8 (206.4-373.6) |
| Vitamin B12 (mcg) | 4.0 (2.2-9.5) |
| Vitamin C (mg) | 47.0 (18.0-88.3) |
| Vitamin D (mg) | 3.7 (1.9-6.9) |

**Supplementary Table 3.** There were relatively few differentially abundant bacterial taxa comparing highest vs. lowest quartile of components of short-term dietary intake (above vs. below median for red meat). There were no differentially abundant taxa associated with intake of other nutrients.

|  | **Family** | **Genus** | **Species** | **Log2Fold Change** | **p** | **Adj p** |
| --- | --- | --- | --- | --- | --- | --- |
| Fruits and vegetables | *Neisseriaceae* | *Neisseria* | *oralis* | 21.71 | <0.001 | <0.001 |
|  | *Burkholderiaceae* | *Lautropia* | *--* | 2.73 | <0.001 | 0.045 |
| Red meat | *Gemellaceae* | *--* | *--* | 1.23 | <0.001 | 0.044 |
|  | | | | | | |
| Cholesterol | *Moraxellaceae* | *Moraxella* | *--* | 16.88 |  | 0.001 |
| Vitamin B12 | *Neisseriaceae* | *Neisseria* | *oralis* | 21.78 | <0.001 | <0.001 |
| Vitamin C | *Neisseriaceae* | *Neisseria* | *oralis* | 21.87 | <0.001 | <0.001 |
| Sugars | *Neisseriaceae* | *Neisseria* | *oralis* | 21.30 | <0.001 | <0.001 |
